# Variants in the 3′ End of SLC6A3 in Northwest Han Population with Parkinson's

**DOI:** 10.1155/2019/6452471

**Published:** 2019-09-03

**Authors:** Peiye Chang, Yongwang Fu, Ping Zhao, Chunmei Wang, Mingfang Jiang, Rui Li, Yulin He

**Affiliations:** ^1^Department of Nuclear Medicine, Inner Mongolia Medical University Affiliated Hospital, Hohhot, China; ^2^Department of Hyperbaric Oxygen Treatment, Inner Mongolia Autonomous Region People's Hospital, Hohhot, China; ^3^Department of Cardiology, Inner Mongolia Autonomous Region People's Hospital, Hohhot, China; ^4^Department of Neurology, Affiliated Hospital of Inner Mongolia Medical University, Hohhot, China; ^5^Department of Neurology, Bayannaoer City Hospital, Bayannaoer, China

## Abstract

Parkinson's disease (PD) is one of the most common neurodegenerative disorders in neurology. It is possible that multifactorial and genetic factors are related to its pathogenesis. Recently, there have been reports of SLC6A3 genetic variants leading to PD. However, the role of 3′ end of SLC6A3 in PD is less studied in different ethnic groups. To explore the roles of 3′ end of SLC6A3 in PD development, 17 SNP sites in 3′ end of SLC6A3 were analyzed in 360 PD patients and 392 normal controls of Han population residing in northwest of China. The significant difference of gene type and allele frequencies between the PD and control groups was detected only in rs40184 (*P* = 0.013 and 0.004, respectively; odds ratio 2.529, 95% confidence interval 1.325–4.827). The genotype and allele frequencies of the other 16 SNP sites were not found to be different between the PD group and the control group. rs2550936, rs3776510, and rs429699 were selected to construct the haplotypes; no significant difference was found in a frequency of 5 haplotypes between the PD group and the control group. These results suggest that the SLC6A3 variant in rs40184 A allele may increase the risk of PD in northwest Han population and may be a biomarker of PD.

## 1. Introduction

Parkinson's disease (PD) is a neurodegenerative disease; its prevalence increases with age, and PD influences 1% of the population over 60 years [1, 2]. Due to the high disability rate and long course of illness, PD has seriously impacted the quality of life of middle-aged and elderly people. Worldwide, the burden of medical expenses on Parkinson's disease has risen from 2.5 million patients in 1990 to 6.1 million patients in 2016, almost doubled in 26 years [3]. The detail mechanism of the etiology and pathogenesis of PD is still unknown. The evidence from most studies showed that PD was a complex multifactor disease influenced by environmental and genetic factors [4, 5], in which genetic factors play a critical role [6–8]. Up to now, the genetic susceptibility of Parkinson's disease is mainly focused on the screening of new genes in the PD family and the exploring of susceptible genes in patients. The genetic susceptibility genes in patients are mainly concentrated in the dopamine metabolic system genes, including the catecholamine oxymethyltransferase gene, the monoamine oxidase gene, the dopamine receptor gene, and the dopamine transporter (DAT) gene (gene symbol: SLC6A3), in which the DAT gene plays an essential role in the pathogenesis of PD. DAT is a transmembrane protein which is expressed by the presynaptic dopamine neuron. The main function of DAT is to reuptake dopamine released into the synaptic space and to stop the transmission of information among nerve cells [9, 10]. In addition, neurotoxin 1-methyl-4-phenyl-1,2,3,6-tetrahydropyridine (MPTP) enters into the synapse cell through DAT. Finally, MPTP destroys the black dopaminergic neurons by oxidative stress.

Since PD is characterized by the selective loss of dopaminergic neurons, genes that affect the expression of dopaminergic neurons may become candidate genes for PD. The evidence from genetic studies revealed that variants in SLC6A3 were associated with PD [11–13]. The most extensively studied polymorphism was the variable number of tandem repeats (VNTR) in the 3′untranslated region (UTR) of SLC6A3 gene. However, the results from a number of related studies have not yet reached a consensus on its genetic correlation with PD. Moreover, there are few related studies on other polymorphisms at the 3′ end of SLC6A3 and PD. In order to further investigate the correlation between SLC6A3 and PD in northwest Han population, we performed a variants study in 3′ end of SLC6A3 in 360 PD patients and 392 healthy controls.

## 2. Materials and Methods

### 2.1. Patients

A cohort of 360 Chinese Han PD patients (63.5 ± 10.4 years) were enrolled. All the patients came from the Inner Mongolia Medical University Affiliated Hospital, Hohhot, China, and Bayannaoer City Hospital, Bayannaoer, China, between 2015 and 2018. All patients live in northwest of China and were not related to each other. All patients were examined by experienced neurologists, and the diagnosis of PD was based on clinical criteria [14]. The control group consisted of 392 age- and sex-matched healthy persons (63.7 ± 9.7 years) from the same geographic areas. This study was approved by the Institutional Review Board of the Inner Mongolia Medical University, Hohhot, China. The study obtained the informed consent of all participants.

### 2.2. Selection of Single-Nucleotide Polymorphisms (SNPs)

TagSNPs were selected from the Chinese HapMap database (http://www.hapmap.org), which is based on pairwise *r*^2^ ≥ 0.8 and minor allele frequency (MAF) ≥0.1. In this study, we chose 17 tagSNPs (rs2270913, rs27048, rs2270914, rs2550936, rs11133767, rs3776510, rs429699, rs11564759, rs27047, rs6347, rs40184, rs37022, rs10036478, rs2652514, rs2981359, rs365663 and rs11133770) from the 3′ end of SLC6A3.

### 2.3. Genotyping

Genomic DNA was extracted from leukocytes in a peripheral blood sample using a blood DNA extraction kit (TIANamp Blood DNA kit; TIANGEN BIOTECH, Beijing, China), which was stored at −20°C. Gene typing was performed using the polymerase chain reaction (PCR)/ligase detection reaction assay. Primers were synthesized by HAYU Biological Engineering LTD in Shanghai; the information of the primers is shown in Table 1. The probe for each group of ligase detection reactions consists of one common probe and two discriminating probes for the two types, as shown in Table 2.

The multiplex PCR methods were used to amplify the target DNA sequences. The final volume of PCRs for each subject was 20 *μ*l, which contained 1X PCR buffer, 3.0 mM/L·MgCl_2_, 2.0 mM/L deoxynucleotide triphosphate, 0.5 *μ*mol/*μ*l primer mix, 5 U/*μ*l Qiagen HotStarTaq Polymerase (QIAGEN, Shenzhen, China), 1X Q-solution, and 50 ng/*μ*l genomic DNA. The thermal cycle was carried out in GeneAmp PCR system 9600 (Norwalk, CT.06859, USA). The initial denaturation was 2 min at 95°C, followed by 40 cycles of denaturation at 94°C for 30 s, annealing at 56°C for 90 s, and extension at 72°C for 1 min. The final extension of 72°C is 10 min.

The total volume of the ligase detection reaction is 10 *μ*l for each subject, which contains 1X NEB Taq DNA ligase buffer 1 *μ*l, 2 pmol of each probe mix 1 *μ*l, 5 U/*μ*l Taq DNA ligase 0.05 *μ*l (BIOWING, Jiangsu, China), and 4 *μ*l multi-PCR product. A total of 40 ligase detection reaction cycles were performed under conditions of 92°C for 2 min, 94°C for 15 s, and 50°C for 25 s. The fluorescent products of ligase detection reaction were identified by PRISM 3730 (ABI). The experimental methods are similar to Chang et al. [15].

### 2.4. Statistical Analysis

Statistical analysis was carried out by the Statistical Program for Social Sciences (SPSS version 11.0). Hardy-Weinberg equilibrium of each group was determined by using the chi-squared test. Allele and genotype frequencies between groups were studied using SHEsis software [16]. We use SHEsis software to calculate the coefficient D′ of linkage disequilibrium (LD) and to build haplotypes. A haplotype with a frequency of less than 3% is considered rare and ignored. When D′ is more than 0.8, it is considered that there is a strong linkage disequilibrium. A *P* value of 0.05 was considered to have statistical significance.

## 3. Results

### 3.1. Association Study of TagSNPs and PD

The genotype and allele frequencies of the 3′ end of SLC6A3 are summarized in Table 3. No deviation from Hardy–Weinberg equilibrium was evident in the PD and control groups (*P* > 0.05). Statistically significant differences in genotype and allele frequencies were found in the SLC6A3 variant rs40184 between PD (AA 5.6%, AG 47.2%, and GG 47.2%) and controls (AA 2.0%, AG 24.0%, and GG 74.0%). The frequency of the minor A allele was 29.2% in patients and 14.0% in controls. There was no significant difference in genotype and allele frequencies for other 16 SNP polymorphism sites in the 3′ end of SLC6A3.

### 3.2. Haplotypes of TagSNPs

**Figure 1:**
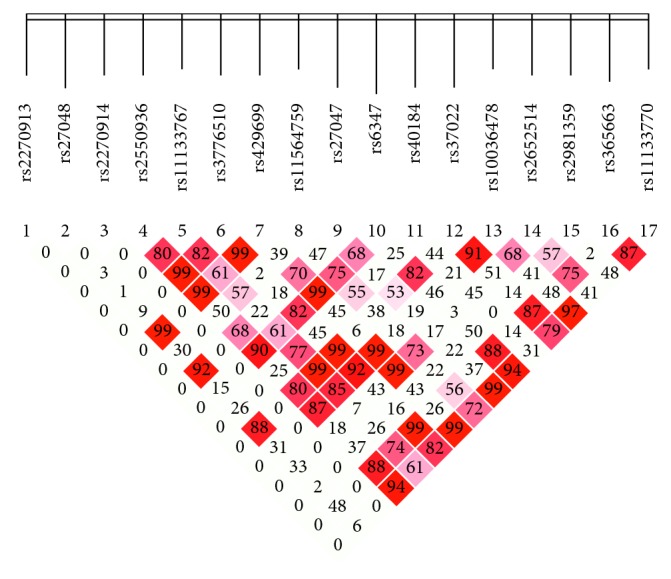
Linkage disequilibrium plots for the 3′ end of SLC6A3.

LD plots of the SLC6A3 in the study are shown in Figure 1. The LD was measured among the tagSNPs by the Lewontin standardized disequilibrium coefficient D′ [17]. Adjacent SNPs in strong LD (D′ > 0.8), rs2550936, rs3776510, and rs429699 have strong LD (D′ > 0.99), which were chosen to build the haplotypes for subsequent analyses. A total of 5 haplotypes were formed, and the frequencies of haplotypes are shown in Table 4. No significant difference was found in frequencies of 5 haplotypes between PD and control groups in the Han population.

## 4. Discussion

PD is a complex disease caused by age and environmental and genetic factors. Finding the genetic susceptibility factors for PD may help identify the individuals at risk and design more specific prevention or treatment options for them. SLC6A3 gene encodes DAT, which comprises 15 exons spanning 60 kb on chromosome 5p15.32 [18]. The studies of the coding region of SLC6A3 have shown that the gene was highly conservative [19]; therefore, the researchers turned the perspective to the noncoding area. The correlation between 40-bp VNTR polymorphisms in the 3′ UTR of SLC6A3 and PD has been studied extensively because VNTR polymorphisms may regulate gene transcription and affect the reuptake of dopamine in the synaptic cleft [20]. The variable numbers of the 40-bp repeat range from 3 to 13 copies; the most common alleles in human beings are the 9- and 10-repeat allele. A lot of studies have shown the association between VNTR polymorphisms of the SLC6A3 and PD in different populations, but the results are inconsistent [21–24]. Two variants, rs28363170 and rs3836790 in SLC6A3, were found to be significantly correlated with PD patients in French population [25], while the variation of these two sites were not related to PD in Han population [26].

Here, we conducted a case-control study of 752 participants in the northwest Han population to further investigate the role of the 3′ end of SLC6A3 in the development of PD. We found that only the SLC6A3 variant rs40184 had statistically significant differences in genotypes and alleles, which may be related to PD. The minor A allele of rs40184 may lead to an increased risk of PD in northwest Han Chinese, and it may be a marker of PD. However, the other 16 polymorphic sites were not found to be related to PD. The reason why the other 16 sites had negative results may be the insufficient number of samples in this study and that these 16 sites may be rare genotypes or alleles in the Han population. Haplotype analysis is thought to be much more powerful than single-nucleotide polymorphism sites in correlation studies [27]. Haplotype analysis greatly reduces the number of test samples and control type I errors although this method will increase the incidence of unavoidable type II errors [28, 29]. Few studies have reported associations between haplotypes of the 3′ end of SLC6A3 and PD; we built the haplotypes among 3 strong LD tagSNPs, while no difference was found between the PD and the control group.

At present, more than 1,500 genes are known to be closely related to the occurrence of diseases. For a long time, coding areas have been a major research direction of genetic diseases and only a small part of noncoding area has been proved to be a useful component that can help genes to be turned on and off to regulate gene expression. The normal expression of genes cannot be separated from the participation of regulatory elements. Abnormalities in certain regulatory components can also lead to mutations in the corresponding gene-coding regions. Therefore, we can assume that the variant rs40184 in the noncoding area of SLC6A3 may lead to differences in susceptibility to PD in our study.

In summary, our findings showed a link between the 3′ end of SLC6A3 gene variant rs40184 and PD in northwest Han population. Given that different populations are genetically heterogeneous and mutations have a specific population frequency, larger sample size studies are needed to confirm the correlation between the 3′ end of SLC6A3 variant and PD in independent larger cohorts and in different geographical origins. Moreover, functional studies of the 3′ end of SLC6A3 need to be carried out to further understand the role of SLC6A3 in PD. Further research is likely to find genetic variations that are risky or protective of PD, which is important for the prevention and treatment of PD.

## Figures and Tables

**Table 1:** Primer of single-nucleotide polymorphisms of SLC6A3.

Gene	Upstream primer	Downstream primer	PCR length
rs2270913	GGTTCCCCTACCTGTGCTAC	AACAGCTTCATCTCGTTTCCG	129
rs27048	AAAGGCGGAGGAGGTGTTC	CCAAACTGCGTTGACTTTTGG	135
rs2270914	GGTTCCCCTACCTGTGCTAC	AACAGCTTCATCTCGTTTCCG	129
rs2550936	CTCCCAAATAATCACGGGGC	GCAGTTGGGTTCCTTCCACC	124
rs11133767	GAGAGGGTGAGCTCCTGAAG	TGCTTTTTGTCACCTGCAGC	139
rs3776510	ACAGAGGAAGGGAGAAAGTGC	GAGAGGGGCGTGGATTTCTC	139
rs429699	CCTCACGGAGCCTTTTTCAG	TTTGGAGTGCTCATCGAAGC	126
rs11564759	AGCGCCCTTGGGAGTTCATG	CACCCAGGGCAGATCTTCC	131
rs27047	ACAAATCACACACGTCCACAC	CCACGTCTAACCTCACGGG	139
rs6347	GGGTTCTGTTTCAGGGCCAG	GATACCCAGGGTGAGCAGC	118
rs40184	TCTGATCAATACGCCCCAGAG	CCAACACACCCTTGACAGG	140
rs37022	TGCTTGCTTTGACCTTTATGG	CCAGCGCCCACTCTCAGTG	140
rs10036478	TCCCAGTTAGGAGCAGGGAG	GAGCTAAAAGGCCATCCAGC	134
rs2652514	CCAGAACCCAGCCACAGAG	ATAGAGGCCAATGAGGGAGG	138
rs2981359	CAGAGTTTAGGAAAGGGAGGC	CGCAGGCTGTTCTTTGGACC	140
rs365663	GTGAGACGCTGGCCATGTC	TTGCCAACCCTGAGGAACAC	138
rs11133770	ACACCTCTGACCACAGTGTG	AAGCCTGGGTTGTGGTCATC	125

**Table 2:** Probe of single-nucleotide polymorphisms of SLC6A3.

Probe name	Sequence (5′–3′)	LDR length
rs2270913_modify	P-GGGGGTCTAGGGCAGCCGTTTTTTTTTTTTTTTTTTTTTT-FAM	
rs2270913_A	TTTTTTTTTTTTTTTTTTCTCCCTGAGCATGCTGGCCGGGT	81
rs2270913_G	TTTTTTTTTTTTTTTTTTTTCTCCCTGAGCATGCTGGCCGGGC	83
rs27048_modify	P-AGTCTGCCTGCTGGTAGCAGTTTTTTTTTTTTTTTTTTTTTTTT-FAM	
rs27048_C	TTTTTTTTTTTTTTTTTTTTTTCTCGTCCCCTCCACCTCCATCCG	89
rs27048_T	TTTTTTTTTTTTTTTTTTTTTTTTCTCGTCCCCTCCACCTCCATCCA	91
rs2270914_modify	P-GACCCCCGCCCGGCCAGCATTTTTTTTTTTTTTTTTTTTTTTTTTT-FAM	
rs2270914_G	TTTTTTTTTTTTTTTTTTTTTTTTTTGCCTTGGCCCCGGCTGCCCCTAC	95
rs2550936_modify	P-ACGGCCCCCAGACCTCCTGTTTTTTTTTTTTTTTTTTTTTTTTTTTTT-FAM	
rs2550936_A	TTTTTTTTTTTTTTTTTTTTTTTTTTAGGCAAGATCCCTGGGCTCACGT	97
rs2550936_C	TTTTTTTTTTTTTTTTTTTTTTTTTTTTAGGCAAGATCCCTGGGCTCACGG	99
rs11133767_modify	P-GCTGCGGCAGCTCCTGGGGCTTTTTTTTTTTTTTTTTTTTTTTTTTTTTT-FAM	
rs11133767_A	TTTTTTTTTTTTTTTTTTTTTTTTTTTTAACGTGCCTTCCTTCCACTGCCT	101
rs11133767_G	TTTTTTTTTTTTTTTTTTTTTTTTTTTTTTAACGTGCCTTCCTTCCACTGCCC	103
rs3776510_modify	P-GCTTCTCCCCATCTCCCGTGTTTTTTTTTTTTTTTTTTTTTTTTTTTTTTTT-FAM	
rs3776510_C	TTTTTTTTTTTTTTTTTTTTTTTTTTTTTTTGAGGTGCAGGTCGCCAGGGCCG	105
rs3776510_T	TTTTTTTTTTTTTTTTTTTTTTTTTTTTTTTTTGAGGTGCAGGTCGCCAGGGCCA	107
rs429699_modify	P-CCCCCGGACTCACCATAGAATTTTTTTTTTTTTTTTTTTTTTTTTTTTTTTTTT-FAM	
rs429699_A	TTTTTTTTTTTTTTTTTTTTTTTTTTTTTTTTGAGGGTGCCGGCTTGGCTGCCTT	109
rs429699_G	TTTTTTTTTTTTTTTTTTTTTTTTTTTTTTTTTTGAGGGTGCCGGCTTGGCTGCCTC	111
rs11564759_modify	P-GGTCTCATGGGGTCTCGGGGTTTTTTTTTTTTTTTTTTTTTTTTTTTTTTTTTTTT-FAM	
rs11564759_C	TTTTTTTTTTTTTTTTTTTTTTTTTTTTTTTTTTAAGATGCAGATCCTGACTGGGCG	113
rs11564759_T	TTTTTTTTTTTTTTTTTTTTTTTTTTTTTTTTTTTTAAGATGCAGATCCTGACTGGGCA	115
rs27047_modify	P-TGTGCCTGGAAGGCGGAGGTTTTTTTTTTTTTTTTTTTTTTTTTTTTTTTTTTTTTT-FAM	
rs27047_C	TTTTTTTTTTTTTTTTTTTTTTTTTTTTTTTTTTTTTGTGGGAGGACCTCAGCTTCCTCG	117
rs27047_T	TTTTTTTTTTTTTTTTTTTTTTTTTTTTTTTTTTTTTTTGTGGGAGGACCTCAGCTTCCTCA	119
rs6347_modify	P-GAGGACAGAGGGAGCGTGGCTTTTTTTTTTTTTTTTTTTTTTTTTTTTTTTTTTTTTT-FAM	
rs6347_A	TTTTTTTTTTTTTTTTTTTTTTTTTTTTTTTTTTTTTTTTTGAAGAAGACCACGGCCCAGGCT	121
rs6347_G	TTTTTTTTTTTTTTTTTTTTTTTTTTTTTTTTTTTTTTTTTTTGAAGAAGACCACGGCCCAGGCC	123
rs2652514_T	TTTTTTTTTTTTTTTTTTTTTTTTTTTTTTTTTTTTTTTTTTTTTTTTTTTTTTTTTTTTTTTTTTTGAAGGGATCACCAATGTTCTTGGACA	172
rs2981359_modify	P-AAACAGGAGGCAGAGCCAAGCTGCCTTTTTTTTTTTTTTTTTTTTTTTTTTTTTTTTTTTTTTTTTTTTTTTTTTTTTT-FAM	
rs2981359_C	TTTTTTTTTTTTTTTTTTTTTTTTTTTTTTTTTTTTTTTTTTTTTTTTTTTTTTTTTTTTTTTTTTTTTTCTTTCCAAAGCGAAGATAGCCTCTGG	175
rs2981359_G	TTTTTTTTTTTTTTTTTTTTTTTTTTTTTTTTTTTTTTTTTTTTTTTTTTTTTTTTTTTTTTTTTTTTTTTTCTTTCCAAAGCGAAGATAGCCTCTGC	177
rs365663_modify	P-TTAGTGGGGCAGCTCAGCAGTCTTTTTTTTTTTTTTTTTTTTTTTTTTTTTTTTTTTTTTTTTTTTTTTTTTTTTTTTT-FAM	
rs365663_C	TTTTTTTTTTTTTTTTTTTTTTTTTTTTTTTTTTTTTTTTTTTTTTTTTTTTTTTTTTTTTTTTTTTTTTTTTTTATTCATGGCACATGGAGGAAGCACCG	180
rs365663_T	TTTTTTTTTTTTTTTTTTTTTTTTTTTTTTTTTTTTTTTTTTTTTTTTTTTTTTTTTTTTTTTTTTTTTTTTTTTTTATTCATGGCACATGGAGGAAGCACCA	182
rs11133770_modify	P-TGATGGGATCAGTGAGGTGCTTAGCTTTTTTTTTTTTTTTTTTTTTTTTTTTTTTTTTTTTTTTTTTTTTTTTTTTTTT-FAM	
rs11133770_A	TTTTTTTTTTTTTTTTTTTTTTTTTTTTTTTTTTTTTTTTTTTTTTTTTTTTTTTTTTTTTTTTTTTTTTTTTTTTTTTTGGGGAGAGGCTTGGCACTGGTCCCTT	185
rs11133770_C	TTTTTTTTTTTTTTTTTTTTTTTTTTTTTTTTTTTTTTTTTTTTTTTTTTTTTTTTTTTTTTTTTTTTTTTTTTTTTTTTTTGGGGAGAGGCTTGGCACTGGTCCCTG	187

**Table 3:** Frequency distribution of SLC6A3 genotypes and alleles.

Gene	Allele/genotype	PD (*n*)	Control (*n*)	*χ* ^2^	*P*	OR	95% CI
rs2270913	C	698 (1.000)	768 (1.000)				
	C/C	349 (1.000)	384 (1.000)				

rs27048	C	620 (0.912)	652 (0.867)	0.939	0.332	0.585	0.620–4.0483
	T	60 (0.088)	100 (0.133)				
	C/C	280 (0.824)	276 (0.734)				
	C/T	60 (0.176)	100 (0.266)	1.089	0.297	1.691	0.626–4.566

rs2270914	G	700 (1.000)	768 (1.000)				
	G/G	350 (1.000)	384 (1.000)				

rs2550936	A	651 (0.930)	712 (0.908)				
	C	49 (0.070)	72 (0.092)	0.271	0.602	1.315	0.469–3.685
	A/A	301 (0.860)	320 (0.816)				
	A/C	49 (0.140)	72 (0.184)	0.301	0.584	0.350	0.460–3.960

rs11133767	A	31 (0.043)	36 (0.046)				
	G	669 (0.956)	748 (0.954)	0.011	0.914	0.930	0.245–3.539
	A/G	31 (0.089)	36 (0.092)				
	G/G	319 (0.911)	356 (0.908)	0.012	0.914	0.927	0.236–3.640

rs3776510	C	668 (0.954)	744 (0.949)				
	T	32 (0.046)	40 (0.051)	0.074	0.786	1.201	0.321–4.495
	C/C	318 (0.909)	352 (0.898)				
	C/T	32 (0.091)	40 (0.102)	0.078	0.780	1.212	0.314–4.686

rs429699	C	500 (0.714)	592 (0.755)				
	T	200 (0.286)	192 (0.245)	0.452	0.502	0.811	0.440–1.495
	C/C	190 (0.543)	220 (0.561)				
	C/T	120 (0.343)	152 (0.388)				
	T/T	40 (0.114)	20 (0.051)	1.679	0.43		

rs11564759	C	403 (0.576)	443 (0.589)				
	T	297 (0.424)	309 (0.411)	0.076	0.783	0.924	0.531–1.611
	C/C	131 (0.374)	116 (0.309)				
	C/T	141 (0.403)	211 (0.561)				
	T/T	78 (0.223)	49 (0.130)	3.303	0.192		

rs27047	C	450 (0.643)	488 (0.622)				
	T	250 (0.357)	296 (0.378)	0.092	0.762	1.092	0.619–1.926
	C/C	150 (0.429)	160 (0.408)				
	C/T	150 (0.429)	168 (0.429)				
	T/T	50 (0.143)	64 (0.163)	0.094	0.954		

rs6347	A	641 (0.914)	771 (0.920)				
	G	59 (0.086)	67 (0.080)	0.001	0.979	1.013	0.383–2.682
	A/A	291 (0.831)	325 (0.829)				
	A/G	59 (0.169)	67 (0.171)	0.001	0.978	1.014	0.365–2.821

rs40184	A	210 (0.292)	112 (0.140)				
	G	510 (0.708)	688 (0.860)	8.245	0.004^*∗*^	2.529	1.325–4.827
	A/A	20 (0.056)	8 (0.020)				
	A/G	170 (0.472)	96 (0.240)				
	G/G	170 (0.472)	296 (0.740)	8.760	0.013^*∗*^		

rs37022	A	314 (0.431)	402 (0.502)				
	T	406 (0.569)	398 (0.498)	1.337	0.246	0.726	0.422–1.250
	A/A	73 (0.194)	93 (0.232)				
	A/T	168 (0.472)	216 (0.540)				
	T/T	119 (0.333)	91 (0.228)	1.83	0.340		

rs10036478	C	614 (0.853)	704 (0.880)				
	T	106 (0.147)	96 (0.120)	0.507	0.476	0.756	0.3499–1.634
	C/C	262 (0.728)	309 (0.773)				
	C/T	90 (0.250)	86 (0.215)				
	T/T	8 (0.028)	5 (0.012)	0.753	0.686		

rs2652514	C	577 (0.824)	693 (0.866)				
	T	123 (0.176)	107 (0.134)	0.736	0.392	0.722	0.343–1.523
	C/C	238 (0.680)	299 (0.748)				
	C/T	101 (0.289)	95 (0.238)				
	T/T	11 (0.031)	6 (0.015)	0.965	0.617		

rs2981359	C	383 (0.532)	388 (0.485)				
	G	337 (0.468)	412 (0.515)	0.680	0.410	1.255	0.731–2.154
	C/C	96 (0.267)	104 (0.260)				
	C/G	191 (0.530)	180 (0.450)				
	G/G	73 (0.203)	116 (0.290)	1.285	0.526		

rs365663	C	420 (0.583)	517 (0.646)				
	T	300 (0.417)	283 (0.354)	0.862	0.353	0.771	0.444–1.337
	C/C	110 (0.306)	165 (0.412)				
	C/T	200 (0.556)	187 (0.468)				
	T/T	50 (0.139)	48 (0.120)	1.224	0.542		

rs11133770	A	658 (0.914)	737 (0.921)				
	C	62 (0.086)	63 (0.079)	0.052	0.820	0.892	0.332–2.395
	A/A	298 (0.828)	337 (0.843)				
	A/C	62 (0.172)	63 (0.157)	0.056	0.812	0.882	0.314–2.482

OR = odds ratio; CI = confidence interval; *χ*^2^ = Pearson chi-square. ^*∗*^*P* < 0.05.

**Table 4:** SLC6A3 haplotype frequency distribution in the Han population.

Haplotype	PD (*N*%)	Control (*N*%)	*χ* ^2^	*P*	OR	95% CI
A C C	450.10 (0.643)	519.79 (0.663)	0.094	0.759	0.914	0.516∼1.620
A C T	200.20 (0.286)	192.08 (0.245)	0.450	0.502	1.233	0.668∼2.275
C C C	19.59 (0.028)	32.14 (0.041)	0.214	0.644	0.691	0.143∼3.336
C T C	30.10 (0.043)	39.98 (0.051)	0.075	0.784	0.831	0.222∼3.117
C T T	0.01 (0.000)	0.01 (0.000)				
Globe *χ*^2^	0.64					
Fisher P	0.89					

OR = odds ratio; CI = confidence interval; *χ*^2^ = Pearson chi-square. ^*∗*^*P* < 0.05.

## Data Availability

The data used to support the findings of this study are included within the article.
